# Effects of anodic transcranial direct current stimulation combined with physical training on the performance of elite swimmers

**DOI:** 10.3389/fphys.2024.1383491

**Published:** 2024-04-11

**Authors:** Xipeng Yang, Jinlong Wu, Yucheng Tang, Zhanbing Ren

**Affiliations:** ^1^ College of Physical Education, Shenzhen University, Shenzhen, China; ^2^ College of Physical Education, Southwest University, Chongqing, China

**Keywords:** transcranial direct current stimulation, physical training, swimming, athletes, sports performance

## Abstract

**Objective:**

Anodal transcranial direct current stimulation (a-tDCS) has been used to improve athletic performance in various populations; however, its role in improving performance in elite athletes is unclear. This study aimed to investigate the effects of a-tDCS on athletic performance in elite athletes.

**Methods:**

We used a single-blind, randomized controlled experimental design and recruited 24 national-level freestyle swimmers from China. All athletes were randomly divided into two groups; the experimental group underwent a-tDCS (current 2 mA for 20 min) combined with physical training, and the control group underwent a-tDCS sham stimulation combined with physical training. The physical training program was identical in the experimental and control groups. The intervention period was 6 weeks, with five weekly sessions of 110 min each, consisting of 20 min of a-tDCS and 90 min of physical training. Base strength, explosive strength, aerobic endurance, and anaerobic endurance were measured in the athletes before and after the intervention.

**Results:**

The results were as follows. 1) Basic strength: There was a significant improvement in 5RM pull-ups in the experimental and control groups before and after the intervention (*p* < 0.05). 2) Explosive strength: There was a significant improvement in vertical jump and swimming start distance into the water in the experimental and control groups before and after the intervention (*p* < 0.05). 3) Aerobic endurance: There was no significant improvement in the experimental and control groups before and after the intervention. 4) Anaerobic endurance: There was a significant improvement in 400 m running performance in the experimental and control groups before and after the intervention (*p* < 0.05).

**Conclusion:**

Compared to physical training alone, a-tDCS combined with physical training can better improve the athletic performance of high-level swimmers, especially in basic strength, explosive strength, and aerobic endurance.

## 1 Introduction

Transcranial direct current stimulation (tDCS) is a non-invasive neuromodulation technique (Nitsche et al., 2008) in which a weak electric current (up to 4 mA) is applied to the brain area of interest to modulate the excitability of neurons (Bikson et al., 2016). tDCS has the advantages of no adverse effects, low cost, easy portability, and operation (Nitsche and Paulus, 2000). It has been widely used in cognitive neuroscience to understand brain function and treat neurological disorders (Kuo et al., 2014; Enhancing et al., 2015).

In recent years, anodal tDCS (a-tDCS) has received increasing attention in sports because of its ability to modulate neural function and promote athleticism (Summers et al., 2016). Neuronal modulation of tDCS improves athletic performance and is an alternative for improving athletic performance (Angius et al., 2017; Holgado et al., 2019; Lattari et al., 2020). The athletic performance of elite athletes stems from the integration of muscles and nerves, which can be trained and improved with appropriate interventions. These interventions’ core are improvements in neural information processing, such as facilitating sensory input, filtering extraneous stimuli, and decreasing motor response time (Fortes et al., 2022a).

Related studies in this field have generally used a-tDCS to stimulate the athlete’s primary motor cortex (M1), which increases neuronal excitability by raising the neuronal resting potential to the activation threshold to complete transient cortical activation (Bindman et al., 1964; Stagg and Nitsche, 2011). Although the mechanism of action of this technique remains controversial (Giordano et al., 2017), the changes in excitability produced by a-tDCS acting on M1 can be demonstrated by the increase in motor-evoked potentials within the small muscles of the hand and the effect of a-tDCS on movement (Nitsche and Paulus, 2000). However, there is currently inconsistency regarding whether a-tDCS improves athletic performance. Seidel-Marzi and Ragert (2020) found that a-tDCS combined with upper- and lower-extremity training interventions was effective in increasing athletes’ endurance performance and decreasing exercise fatigue, while Anoushiravani et al. (2023) found that a-tDCS combined with gymnastics training was effective in improving athletic performance and motor cognition in athletes. In contrast, some studies did not find that a-tDCS improved athletic performance. For example, Da Silva Machado et al. (2022) found that a-tDCS combined with a cycling intervention failed to improve athletic performance and psychophysiological responses in athletes and suggested that this may be due to a ceiling effect in athletes.

Overall, a-tDCS may lead to shorter reaction times, improved motor accuracy, or delayed fatigue in some specific tasks. However, some studies have failed to replicate these results or found individual differences in their effects. Factors affecting this inconsistency may include differences in the stimulation parameters, individual participant differences, and task characteristics.

Few studies have investigated the effects of a-tDCS on the athletic performance of elite swimmers. Penna et al. (2021) tested ten elite swimmers in 800 m freestyle after tDCS stimulation and found that tDCS stimulation did not affect athletic performance. Nikooharf Salehi et al. (2022) tested 15 professional swimming athletes in 50 m freestyle after tDCS stimulation and found that tDCS stimulation can reduce the negative impact of mental fatigue on swimming performance. The present study used a-tDCS intervention combined with physical training to investigate the effect of this training method on the athletic performance of elite swimmers and to provide more evidence for the role of a-tDCS in improving swimmers’ competitive level.

## 2 Materials and methods

### 2.1 Participants

In this study, we determined the minimum sample size required to ensure that our research had sufficient statistical power to test our hypothesis by conducting a prior power analysis. We employed the G*Power software for power analysis to determine the minimum sample size required to test the main study hypothesis with an alpha level of 0.05 and a power of 0.80. Based on the results of our preliminary study and the effect size of similar interventions reported in the literature, we calculate that at least 20 participants are needed to achieve the desired statistical efficacy. Therefore, we recruited 24 Chinese national elite freestyle swimmers to participate in this experiment. This study used a single-blind, pseudo-randomized controlled trial to test whether a-tdcs combined with physical training is superior to physical training alone in improving athletic performance in high-level swimmers. Twenty-four Chinese national-level elite freestyle swimmers were recruited to participate in this experiment. The 24 elite swimmers were randomly divided into experimental and control groups. The experimental group received a-tDCS true-stimulation intervention combined with physical training, and the control group received a-tDCS sham stimulation combined with physical training.

Inclusion criteria for high-level swimmer participants were (1) no sports injuries in the last 6 months; (2) no psychotropic or sedative drugs in the last 3 months; (3) good physical and mental condition to complete the experimental test; (4) no contraindications to transcranial direct current stimulation, such as skin allergies and metal implants in the body; and (5) no intake of stimulant beverages such as caffeine, alcohol, and other stimulants for 24 h before each test. Table 1 presents the participants’ characteristics.

**TABLE 1:** Basic characteristics of athletes.

	a-tDCS (n = 12)	Sham (n = 12)	P
Gender	Male: 9, Female: 3	Male: 9, Female: 3	0.150
Age (yr)	20.25 ± 1.96	20.33 ± 1.37	0.909
Height (cm)	173.56 ± 5.67	174.43 ± 4.78	0.467
Weight (kg)	68.13 ± 10.12	70.34 ± 8.69	0.534
BMI (kg/m^2^)	22.78 ± 3.97	23.29 ± 2.78	0.621
Years of training (yr)	10.70 ± 1.43	10.17 ± 1.04	0.494

Note: BMI, body mass index.

Heavy exercise was prohibited for 24 h before each test. Before the experiment, all the participants were informed of the purpose of the study, understood the experimental procedures and precautions, agreed to participate voluntarily, and signed an informed consent form. The study was approved by the Medical Ethics Review Committee of Shenzhen University School of Medicine (PN-202200127).

### 2.2 a-tDCS intervention

The a-tDCS was performed using the Halo Sport tDCS device manufactured by Halo Neuroscience, Inc., in the U.S. This instrument has a total weight of less than 2 kg, is easy to carry, and consists of headphones similar to conventional headphones. It delivers a variable DC of up to 2 mA across the scalp via surface electrodes (Brunoni et al., 2012) using 7 cm × 5 cm electrode sheets with a current intensity adjustment range of 0 ∼ 5 mA and a maximum voltage of 20 V (Luo et al., 2023a).

**FIGURE 1:**
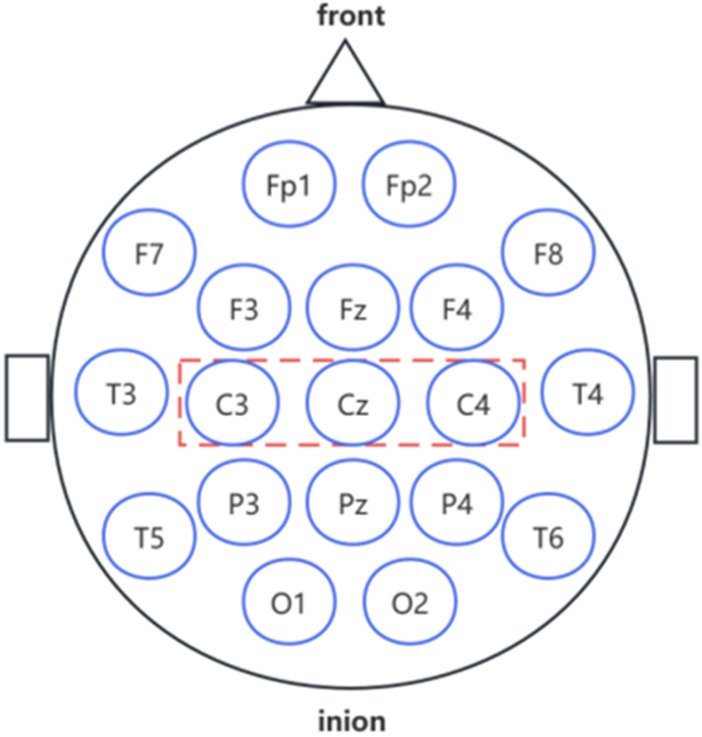
Schematic of the International 10–20 system.

In this study, the bilateral M1 brain region was identified as the main target of a-tDCS to modulate neural excitability and improve motor performance based on the findings of Machado et al. (2019). We selected the bilateral M1 as the brain region of interest to apply a-tDCS. a-tDCS was based on the placement scheme of the International 10–20 EEG System (Figure 1). The three electrode pads of the Halo Sport device were immersed in saline (0.9% NaCl) (Wang et al., 2022). The participants’ scalps were coated with a conductive paste, and the anodes were placed at C3 and C4 of the brain to cover the M1 region. Currently, there is no strict limit to the stimulation time of a-tDCS, but 20 min is generally considered to be optimal, and the stimulation current applied to the person should be less than 2 mA; 1 mA or less is used clinically. If the measurements are repeated, the intervals are generally considered at least 48 h (Alix-Fages et al., 2019).

The intensity and intervention time used in this study were set in advance. The current intensity of the real stimulus gradually increased to 2 mA within 30 s and was maintained at this level for 20 min. The sham stimulus’s initial stimulus intensity and electrode placement position were the same as those of the real stimulus. Still, the intensity was maintained at the beginning of the stimulus phase for 30 s and then decreased to 0 mA, which was used to produce an itchy sensation of tDCS in the scalp area to make the control participants believe that the stimulation was occurring (Kantak et al., 2012). All participants were asked to remain seated and free of verbal body movements during a-tDCS to avoid disturbance by extraneous factors (Nitsche et al., 2008). The apparatus was operated by a single person who had received full training and was not involved in the experiment. Similar stimulation settings have been tested in several clinical trials and are considered safe for humans (Palm et al., 2013).

### 2.3 Physical training programmes

The physical training lasted for 6 weeks, five times a week, for 90 min. The training goal was to develop the swimmer’s basic strength, explosive power, and aerobic and anaerobic endurance. Basic strength training consisted of 10RM pull-ups, 10RM deep squats, 5RM pull-ups, 5RM deep squats, and 10RM snatches of 20 self-weighted pull-ups. Explosive strength training consisted of 15 s pull-ups, 1RM bench press, 10RM seated squat, and 5RM seated squat. Aerobic and anaerobic endurance training consisted of a 3,000 m run, 400 m run, and 200 m run. After 5 days of physical training, 2 days of rest allowed the athletes to regenerate their muscles (Mujika and Crowley, 2019). Table 2 shows the daily programs for the physical fitness interventions.

**TABLE 2:** Specific programs for physical training.

Dates	Physical fitness intervention components
Monday	4–6 sets of 10RM pull-ups, 3–4 sets of 15 s pull-ups, 2–3 sets of 5RM deep squats, 2–3,400 m running
Tuesday	4–6 sets of 1RM bench press, 3–4 sets of 10RM snatch, 2–3 sets of 20 self-weighted pull-ups, 1–2 sets of 3,000 m running
Wednesday	4–6 sets of 10RM deep squats, 3–4 sets of 10RM seated squats, 2–3 sets of 5RM pull-ups, 2–3 sets of 400 m running
Thursday	2–3 sets of 20 self-weighted pull-ups, 3–4 sets of 10RM snatches, 4–6 sets of 1RM bench presses, 1–2 sets of 3,000 m running
Friday	2–3 sets of 5RM pull-ups, 3–4 sets of 5RM seated squats, 2–3 sets of 5RM deep squats, 2–3 sets of 200 m running
Saturday	Breaks
Sunday	Breaks

Note: RM, repetition maximum.

### 2.4 Experimental procedure

After the 24 athletes met the inclusion criteria and signed an informed consent form, the researchers explained the experimental procedures and precautions. To control and document any adverse reactions caused by tDCS, participants were asked to complete a standardized adverse reaction questionnaire after each stimulus. The questionnaire is designed to capture changes in mood, skin sensations, headaches, or any other discomfort associated with tDCS.The researchers tested all athletes on physical fitness and swimming-related sports performance parameters. All test items were preceded by a warm-up session, including a 10-min rowing machine warm-up before the physical fitness parameter test and a 400 m freestyle and four 15 m freestyle warm-ups for the swimming-related sports performance test. Each participant was tested twice for each parameter, and the best score was used for analysis.

**FIGURE 2:**
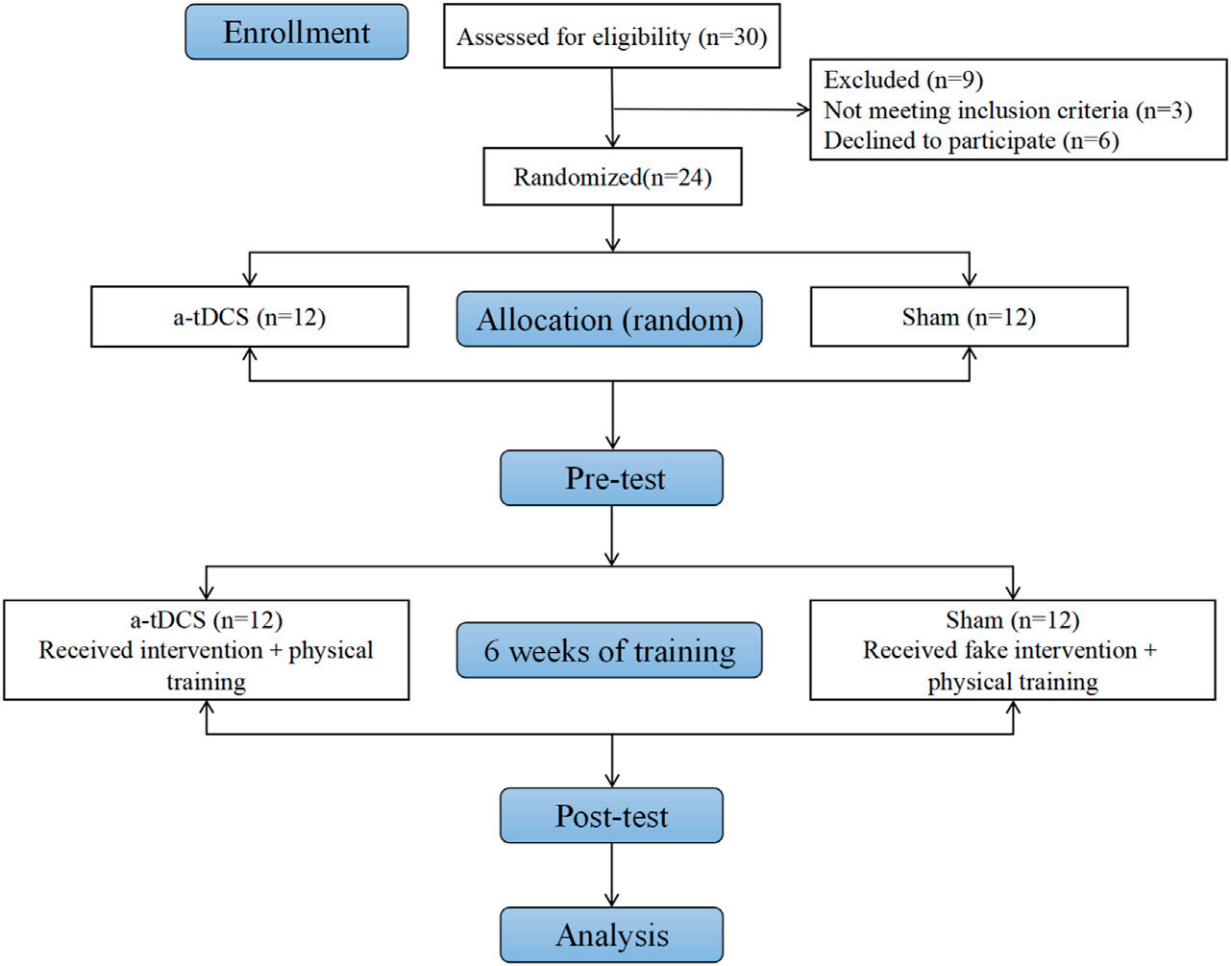
Flow chart of the experiment.

After the pretest, the two groups of athletes immediately participated in a-tDCS combined with physical intervention training, 110 min each time, five times a week for 6 weeks. The intensity of training was controlled by the Rating of Perceived Pain (RPE) scale of 9. At the end of each training, the trained muscle group was rolled on the foam axis and static traction for about 10–15 min. After 6 weeks of intervention training, the athletes resumed training. After the Fatigue Scale-14 (FS-14), it was found that the fatigue degree of each athlete was relieved enough. The same process as the pre-test was carried out. The flow chart is shown in Figure 2.

### 2.5 Athletic performance assessment

This study, 12 test parameters were set up with swimming characteristics, including four aspects of basic strength, explosive strength, aerobic endurance, and anaerobic endurance.

#### 2.5.1 Basic strength

(1) Self-weighted pull-ups: Athletes were required to complete the maximum number of pull-ups without wobbling (uniformly using an assisted band to avoid the uncertainties associated with grip strength gaps). (2) 5RM Pull-ups: Athletes were required to use a pull-up weighted belt and add weight until they could only complete five standard pull-ups (All athletes use the same type of auxiliary strap, which makes it easier for athletes to grip the horizontal bar, to avoid uncertainty due to the gap in grip strength). (3) Self-supporting squat: Athletes were required to use a squat rack and a barbell and squat with their body weight until the maximum number of repetitions was achieved. (4) 5RM Squat: Athletes were required to use a squat rack and a barbell, and squat weight was added until they could only complete five standard squats.

#### 2.5.2 Explosiveness

(1) 15 s pull-up: Athletes were allowed to complete body-swaying pull-ups with a maximum number of repetitions in 15 s (All athletes use the same type of auxiliary strap, which makes it easier for athletes to grip the horizontal bar to avoid uncertainty due to the gap in grip strength). (2) Vertical jump: This test used a feeling tape and tape measure and required the athlete to jump with both feet, feel the height with one hand, and strike the feeling tape with the hand (no running aid was allowed, and only one swing of the arm was allowed). (3) Swimming starting distance: The test used a standard 50 m swimming pool and a high-definition camera to calculate the starting distance through the 5 m line mark at the bottom of the pool (when athletes performed the 25 m freestyle test, the athletes’ starting distance was measured at the same time to make the test data more realistic). (4) 25 m freestyle: The test was conducted using a standard 50 m swimming pool and a stopwatch with manual timing, and obvious markings were set at the bottom of the 25 m pool and the waterline, respectively (to avoid experimental errors, one-on-one timing was conducted by the testers).

#### 2.5.3 Aerobic endurance

(1) 1,000 m run: The test used an electronic timer, and each athlete carried a timing sensor on their body, which required the athlete to complete the 1,000 m run as fast as possible (during the test, athletes were allowed to merge). (2) 400 m freestyle: The test was manually timed using a standard 50 m swimming pool and a stopwatch (to avoid experimental errors, the testers were timed in pairs of four).

#### 2.5.4 Anaerobic endurance

(1) 400 m run: The test used an electronic timer, and each athlete carried a timing sensor on their body, which required the athlete to complete the 400 m run as fast as possible (during the test, athletes were not allowed to merge). (2) 200 m freestyle: The test was manually timed using a standard 50 m swimming pool and a stopwatch (to avoid experimental errors, the testers were timed in pairs of four).

### 2.6 Data analysis

The valid data on exercise performance parameters were collected two times, including physical fitness and swimming-related exercise performance. The mean value of the valid data was taken for statistical analysis, and all parameter values were expressed as “mean ± standard deviation” (mean ± SD). Two-factor repeated measures analysis of variance (ANOVA) with IBM SPSS Statistics 27 statistical analysis software was used to test the interaction between stimulus type (true stimulus, sham stimulus) and time (before intervention, after intervention). If any interaction was significant, it was judged as a simple main effect. A paired-sample *t*-test was used to analyze the differences in indicators related to the athletic performance of swimmers before and after stimulation, and the significance level was set at 0.05.

## 3 Results

### 3.1 Basic strength

**FIGURE 3:**
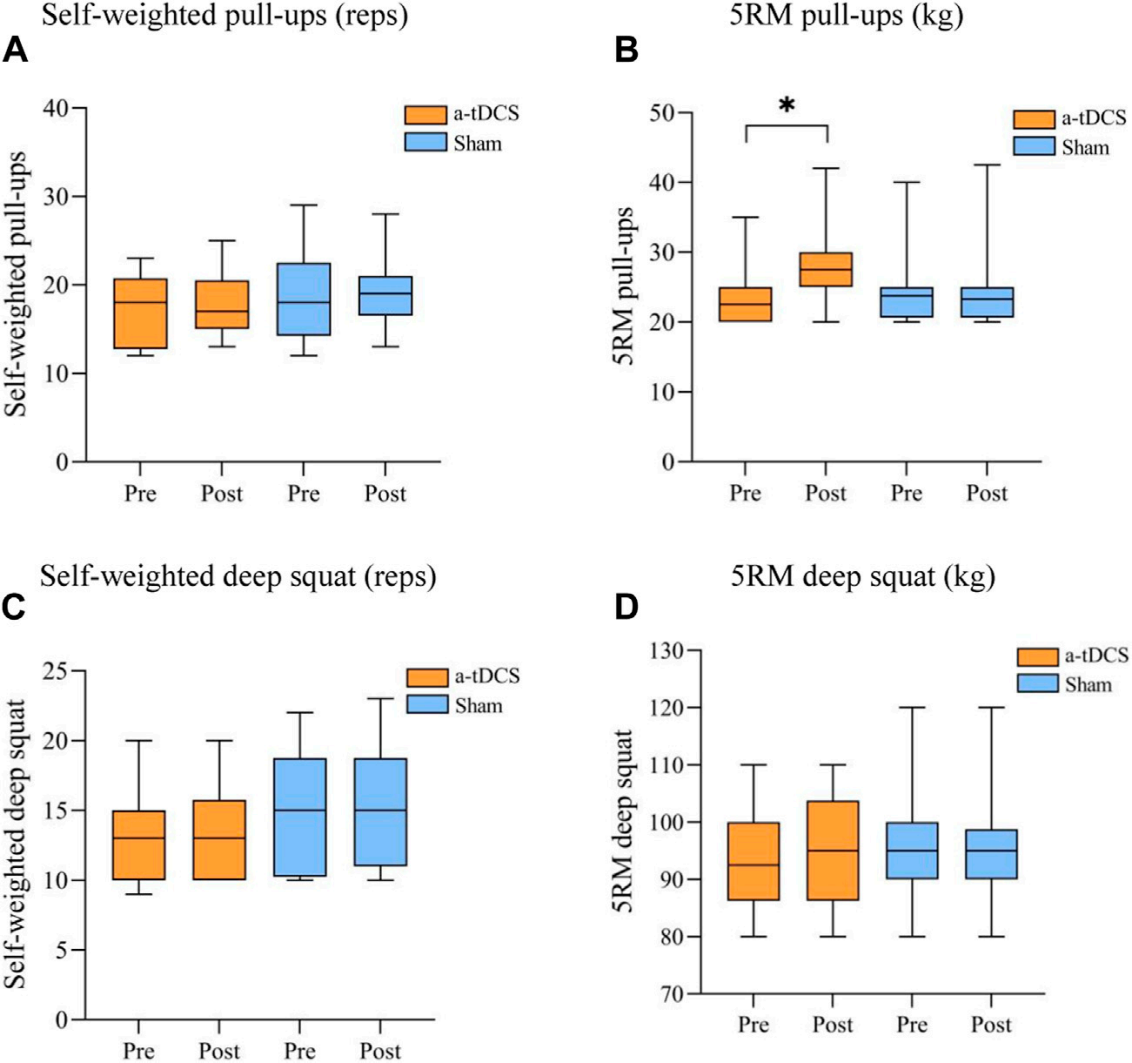
Comparison of the performance of the participant athletes before and after the basic strength intervention. **(A)** Self-weighted pull-ups (reps), **(B)** 5RM pull-ups (kg), **(C)** Self-weighted deep squat (reps) **(D)** 5RM deep squat (kg). Note: * indicates a significant difference between pre- and post-stimulation (*p* < 0.05), RM, repetition maximum.

Table 3 demonstrates the results of a repeated-measures ANOVA of basic strength before and after the intervention for both groups of athletes. W found a main effect of time factor for 5RM pull-ups [P(time) = 0.080)] and an interaction between the type of stimulus and before and after the stimulus [F(1, 22) = 15.052, P(time ×intervention mode) = 0.031]. A simple effects analysis showed that, compared to the control group, there was a significant improvement in 5RM pull-ups before and after intervention in the experimental group (*p* = 0.015, Figure 3B). For self-weighted pull-ups [F(1, 22) = 0.401, P(time × intervention mode) = 0.533, P(time) = 0.650, P(intervention mode) = 0.246], Self-weighted deep squat [F(1, 22) = 0.234, P(time × intervention mode) = 0.633, P(time) = 0.861, P(intervention mode) = 0.348], and 5RM deep squat experimental group [F(1, 22) = 1.599, P(time × intervention mode) = 0.219, P(time) = 0.884, P(intervention mode) = 1.000], there were no interactions between stimulus type and pre- and post-stimulation (Figures 3A, C, D).

**TABLE 3:** Changes in basal strength before and after the intervention in both groups of athletes.

	a-tDCS (n = 12)		Sham(n = 12)		F	P (time × intervention mode)	P(time)	P(intervention mode)
	Pre	Post	Pre	Post				
Self-weighted pull-ups (reps)	17.35 ± 4.38	17.67 ± 3.87	18.58 ± 4.96	19.42 ± 4.40	0.401	0.533	0.650	0.246
5RM pull-ups (kg)	23.75 ± 4.71	28.48 ± 4.76*	24.79 ± 5.59	24.91 ± 5.13	15.141	0.031#	0.080×	0.312
Self-weighted deep squat (reps)	13.33 ± 3.92	13.58 ± 3.80	14.50 ± 4.30	14.67 ± 4.38	0.234	0.633	0.861	0.348
5RM deep squat (kg)	93.75 ± 8.82	95.00 ± 9.29	94.58 ± 10.54	94.17 ± 10.41	1.599	0.219	0.884	1.000

Note: * indicates a significant difference between pre- and post-stimulation (*p* < 0.05), ** indicates a highly significant difference between pre- and post-stimulation (*p* < 0.01), # indicates an interaction between stimulus type and pre- and post-stimulation (*p* < 0.05), ## indicates a strong interaction between stimulus type and pre- and post-stimulation (*p* < 0.01), RM, repetition maximum.

### 3.2 Explosiveness

**FIGURE 4:**
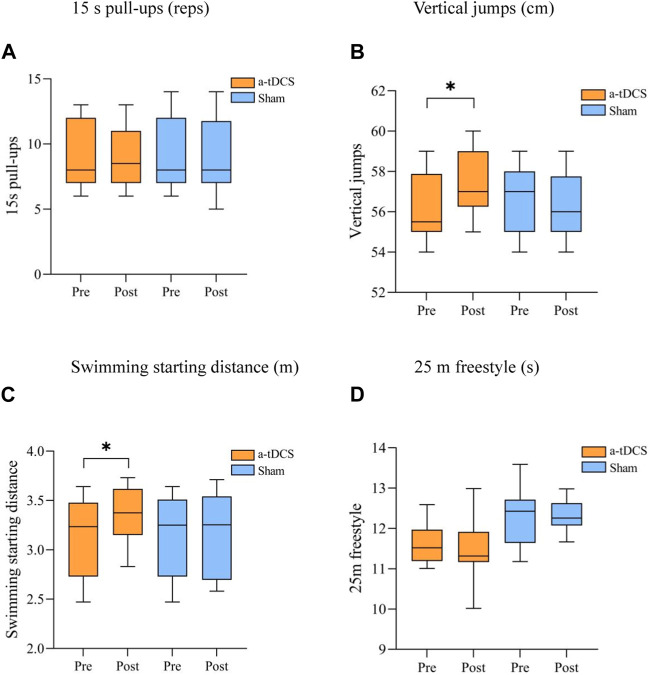
Comparison of the performance of the participant athletes before and after the explosive power intervention. **(A)** 15 s pull-ups (reps) **(B)** Vertical jumps (cm), **(C)** Swimming starting distance (m) **(D)** 25 m freestyle (s). Note: * indicates a significant difference between pre- and post-stimulation (*p* < 0.05).

Table 4 demonstrates the results of the repeated-measures ANOVA for explosive power before and after the intervention for both groups of athletes. We found a strong main effect of the time factor for Vertical jumps [P(time) = 0.009] and a strong interaction between stimulus type and before and after the stimulus [F(1, 22) = 13.613, P(time × intervention mode) = 0.001]. A simple effects analysis showed that, compared to the control group, there was a significant improvement in Vertical jumps before and after the intervention in the experimental group (*p* = 0.026, Figure 4B). There was a main effect of the time factor for swimming starting distance into the water (P [time] = 0.04) and the interaction between the type of stimulus and the pre- and post-stimulus [F(1, 22) = 5.452, P(time × intervention mode) = 0.029]. Simple effects analyses showed a significant increase in the swimming starting distance before and after the intervention in the experimental group compared to the control group (*p* = 0.043, Figure 4C); 15 s pull-ups [F(1, 22) = 8.290, P(time × intervention mode) = 0.322, P(time) = 0.110, P(intervention mode) = 0.565] and 25 m freestyle [F(1, 22) = 8.409, P(time × intervention mode) = 0.220, P(time) = 0.217, P(intervention mode) = 0.753] stimulus type did not interact with either pre- or post-stimulation (Figures 4A, D).

**TABLE 4:** Changes in explosive power before and after intervention in both groups of athletes.

	a-tDCS (n = 12)		Sham (n = 12)		F	P (time ×intervention mode)	P(time)	P(intervention mode)
	Pre	Post	Pre	Post				
15s pull-ups (reps)	9.08 ± 2.78	9.17 ± 2.62	9.42 ± 2.77	9.52 ± 2.59	8.352	0.322	0.110	0.565
Vertical jumps (cm)	56.25 ± 0.67	57.98 ± 0.98*	56.72 ± 1.11	56.19 ± 1.33	13.313	0.013#	0.029×	0.847
Swimming starting distance (m)	3.13 ± 0.38	3.37 ± 0.22*	3.14 ± 0.42	3.15 ± 0.48	5.432	0.029#	0.040×	0.283
25 m freestyle (s)	11.63 ± 0.56	11.47 ± 0.71	12.31 ± 0.69	12.32 ± 0.74	8.329	0.220	0.217	0.753

Note: * indicates a significant difference between pre- and post-stimulation (*p* < 0.05), ** indicates a highly significant difference between pre- and post-stimulation (*p* < 0.01), # indicates an interaction between stimulus type and pre- and post-stimulation (*p* < 0.05), ## indicates a strong interaction between stimulus type and pre- and post-stimulation (*p* < 0.01).

### 3.3 Aerobic and anaerobic endurance

**FIGURE 5:**
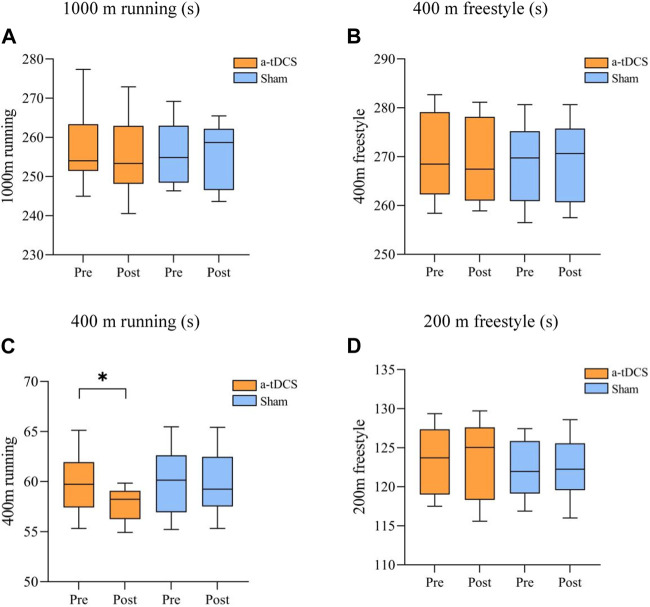
Comparison of swimmers’ performance before and after aerobic and anaerobic endurance interventions. **(A)** 1,000 m running (s) **(B)** 400 m freestyle (s), **(C)** 400 m running (s) **(D)** 200 m freestyle (s). Note: * indicates a significant difference between pre- and post-stimulation (*p* < 0.05).

Table 5 demonstrates the results of the repeated-measures ANOVA for aerobic and anaerobic endurance before and after the intervention for both groups of athletes. We found a time factor main effect for 400 m running [P(time) = 0.037] and an interaction between the type of stimulus and the before and after of the stimulus [F(1, 22) = 4.792, P(time × intervention mode) = 0.040]. A simple effects analysis showed a significant increase in 400 m running performance before and after intervention in the experimental group compared to the control group (*p* = 0.0164, Figure 5C) and 1,000 m running [F(1, 22) = 0.026, P(time × intervention mode) = 0.874, P(time) = 0.750, P(intervention mode) = 0.786], 400 m freestyle [F(1, 22) = 3.227, P(time × intervention mode) = 0.086, P(time) = 0.422, P(intervention mode) = 0.369], 200 m freestyle [F(1, 22) = 9.183, P(time × intervention mode) = 0.304, P(time) = 0.199, P(intervention mode) = 0.952]. There was no interaction between the stimulus type and the pre- and post-stimulus (Figures 5A, B, D).

**TABLE 5:** Changes in aerobic and anaerobic endurance capacity before and after intervention in both groups of athletes.

	a-tDCS (n = 12)		Sham (n = 12)		F	P(time ×intervention mode)	P(time)	P(intervention mode)
	Pre	Post	Pre	Post				
1000 m running (s)	257.12 ± 9.19	255.23 ± 9.37	256.2 ± 7.59	255.62 ± 7.35	0.026	0.874	0.750	0.786
400 m freestyle (s)	270.11 ± 8.77	269.27 ± 8.45	269.03 ± 8.00	269.36 ± 8.47	3.227	0.874	0.422	0.369
400 m running (s)	59.80 ± 2.89	57.83 ± 2.34*	60.12 ± 3.11	59.98 ± 2.96	4.792	0.040#	0.037×	0.121
200 m freestyle (s)	123.41 ± 4.26	123.42 ± 4.13	122.23 ± 3.72	122.51 ± 3.57	9.213	0.304	0.199	0.952

Note: * indicates a significant difference between pre- and post-stimulation (*p* < 0.05), ** indicates a highly significant difference between pre- and post-stimulation (*p* < 0.01), # indicates an interaction between stimulus type and pre- and post-stimulation (*p* < 0.05), ## indicates a strong interaction between stimulus type and pre- and post-stimulation (*p* < 0.01).

## 4 Discussion

In this study, we used a randomised controlled trial to test the superiority of 6 weeks of a-tDCS technique combined with physical training over physical training alone in improving the athletic performance of high-level swimmers. Specifically, our results showed that a-tDCS combined with physical training improved basic strength, explosiveness, and anaerobic endurance in swimmers, with no significant differences found in aerobic endurance.

Basic strength refers to the muscular strength of swimmers, which is the basis of the power required for swimming starts, turns, and movements. An improvement in muscular strength can effectively improve athletic performance (Keiner et al., 2019). In this study, self-weighted pull-ups, self-weighted deep squats, 5RM pull-ups, and 5RM deep squats were used to test the basic strength levels of high-level swimmers. The results showed that a-tDCS had a significant effect on swimmers’ 5RM pull-ups, whereas there was no significant difference between pre- and post-intervention for self-weighted pull-ups, self-weighted deep squats, and 5RM deep squats.

This result is similar to that of other studies. Kan et al. tested the maximal voluntary isometric contraction (MVC) of the biceps brachii muscle of the upper limb, and they found no significant difference in MVC intensity between the first and second a-tDCS (Kan et al., 2013). Kazuhei et al. performed a-tDCS lower-limb muscle strength training on participants, and they found the peak torque in knee extension and flexion increased significantly in both groups after the intervention, with no statistically significant difference between the anodal and sham tDCS groups (Kazuhei et al., 2017). However, Hendy et al. studied the effect of 3 weeks of a-tDCS intervention on upper limb strength training. The experimental results showed that a-tDCS combined with physical training and a-tDCS alone improved by 17.29% and 15.15%, respectively. In contrast, there was no significant improvement in sham a-tDCS combined with physical training (Kan et al., 2013). Therefore, we inferred that the possible mechanism for the significance of the 5RM pull-ups is the effect of a-tDCS on improving upper limb back-base strength, which can improve cortical spinal cord excitability (Hendy and Kidgell, 2014).

Short-distance swimming events are highly demanding in terms of explosive power, and swimmers usually do not have sufficient time to maximize power in short-distance events, where success often depends on the rate of power development (Janz and Malone, 2008). In this study, a 15 s pull-up, vertical jump, swimming starting distance, and 25 m freestyle test were used to assess the explosive power of swimmers, and the results showed that a-tDCS had a significant effect on swimmers’ vertical jump and swimming starting distance into the water.

This result is consistent with the results of national and international studies. Grandperrin et al. tested participants for vertical jump explosiveness and found that a-tDCS intervention increased the vertical jumping ability of athletes (Grandperrin et al., 2020). Luo et al. tested 92 rock climbers with 9 min of incremental aerobic exercise and single-arm pull-down and found that a-tDCS improved the single-arm pull-down explosive power in rock climbers (Luo et al., 2023b). Lu et al. randomly assigned 20 healthy men to an a-tDCS intervention and measured surface electromyography of the rectus femoris and biceps femoris, which showed that the diameter of the rectus femoris and biceps femoris was significantly higher after real stimulation than after sham stimulation (Lu et al., 2021). Thng et al. explored how to improve the level of swimming departure found that swimming departure performance was almost perfectly correlated (r > 0.90) with vertical jump height (Thng et al., 2019); therefore, the present study also included the level of swimming departure in the explosive strength level test. We speculate that adding a-tDC stimulation to traditional physical training could increase the number of recruitments per muscle unit and thus enhance athletic performance (Janz and Malone, 2008).

Aerobic and anaerobic capacities are the key abilities for long-distance swimming. Currently, regarding the methods for improving aerobic and anaerobic endurance in professional swimmers, most coaches and sports researchers choose to create low-pressure hypoxic environments for athletes, thus increasing their blood oxygen concentrations and enhancing cardiorespiratory fitness (Bonne et al., 2014). In this study, 1,000 m running and 400 m freestyle tests were used to respond to the aerobic endurance level of swimmers, and 400 m running and 200 m freestyle tests were used to respond to the anaerobic endurance level of swimmers. The results showed that a-tDCS significantly improved 400 m running in swimmers. This result is consistent with previous national and international studies. Mesquita et al. conducted aerobic tests on taekwondo athletes and found that a-tDCS did not significantly affect the aerobic performance of professional taekwondo athletes (Mesquita et al., 2020). Judge et al. explored whether a-tDCS could improve cycling endurance performance in the general population by experimenting with a 10-min fixed-cycle intensity and a 15-min self-paced time trial and showed that a-tDCS could not improve exercise endurance (Judge et al., 2020). Zhiqiang et al. tested a-tDCS intervention on eight rowers from a national team for 5 km rowing and found that the a-tDCS technique was unable to improve the aerobic endurance of the rowers; it was hypothesized that the mechanism of the inability of a-tDCS to affect the aerobic endurance of the swimmers might be the inability of a-tDCS to slow down the sensation of fatigue of the swimmers for long-distance exercise. It may be possible that only the neurophysiological mechanisms of the brain activities related to endurance can be improved using electroencephalogram and magnetic resonance imaging to perfect the research in this field (Zhiqiang et al., 2022).

There is limited relevant literature on whether a-tDCS improves anaerobic endurance; however, the results are relatively uniform. Fortes et al. explored the idea that a-tDCS could maintain endurance performance in mentally fatigued swimmers in an experiment in which athletes were given a 3-min full-speed swim, similar to performing an anaerobic endurance test. The results showed that a-tDCS helped swimmers improve their anaerobic endurance during competitive events (Fortes et al., 2022b). Sasada et al. found that a-tDCS delayed the feeling of fatigue in participants’ medium- and long-distance cycling, thus increasing the athletes’ anaerobic endurance. It was hypothesized that the mechanism by which a-tDCS was effective in increasing anaerobic endurance in swimmers was that a-tDCS could delay the feeling of fatigue produced by medium- and long-distance exercise in swimmers, thus increasing anaerobic endurance (Sasada et al., 2020).

The present study found that a-tDCS significantly improved anaerobic endurance in swimmers, as evidenced by the significant variability before and after the 400 m running stimulus. Therefore, we hypothesized that this technique could improve the athletic performance of middle- and long-distance track and field athletes and runners.

Our study has some limitations. First, the elite athletes we included were only freestyle swimmers, and whether the findings can be generalized to other sports needs further confirmation. In addition, with the development of tDCS technology, more advanced stimulation instruments may show better aggregation and stimulation effects. Third, muscle recruitment was not further tested in this study; therefore, further studies are needed to determine muscle recruitment and to elucidate the underlying mechanisms of a-tDCS. Fourth, this study only explored the effects of a-tDCS on the athletic performance of elite swimmers and did not explore the effects of the technique on swimmers at different skill levels. Fifth, this study only performed stimulation of the C3 and C4 cortical regions of swimmers and did not explore whether stimulation of other brain regions had any effect on locomotor performance.

## 5 Conclusion

The experimental findings from this study preliminarily validate that a regimen combining 20 min of anodal transcranial Direct Current Stimulation (a-tDCS) at 2 mA with targeted physical fitness training for swimmers can markedly enhance their foundational strength, explosive power, and anaerobic endurance. This is evidenced by improvements across several key performance indicators: an increase in the number of repetitions for 5 Repetition Maximum (5RM) pull-ups, a noticeable enhancement in vertical jump height, improvements in the distance achieved during the swimming start, and better performance times in the 400-m run. a-tDCS is an effective means of auxiliary sports training, can improve the swimmer’s sports performance, and has application value for increasing athletes’ sports potential. Elite swimmers can consider the a-tDCS technology applied in energy and swimming training to improve the swimmer’s comprehensive sports level.

## Data Availability

The raw data supporting the conclusion of this article will be made available by the authors, without undue reservation.
